# Unveiling the toxicological and biochemical evaluation of selected botanical extracts against adult *Aphis gossypii* (Aphididae: Hemiptera)

**DOI:** 10.1038/s41598-026-66584-0

**Published:** 2026-08-21

**Authors:** Abdel Fattah Khalaf, Karam Hussein, Shehta Ali, Doaa Barakat, Mohamed Gad

**Affiliations:** 1 Department of Zoology, Faculty of Science, Zagazig University, Zagazig, Egypt; 2 Plant Protection Research Institute, Agricultural Research Center, Giza, Egypt

**Keywords:** *Aphis gossypii*, Botanical extracts, Fixed oils, Toxicity, Sub-lethal effects, Biochemical effects, LC_50_, Biochemistry, Biological techniques, Chemical biology, Environmental sciences, Plant sciences

## Abstract

The present study evaluated the toxicity and biochemical effects of selected botanical extracts against the adult stage of *Aphis gossypii*. Two fixed oils, namely *Croton tiglium* seed oil and *Simmondsia chinensis* seed oil, in addition to ethanolic extracts of *Urtica dioica* and *Hyoscyamus muticus*, were assessed for their insecticidal activity under laboratory conditions. Toxicity was determined based on LC_50_ values after 48 h of exposure. The results revealed that *C. tiglium* seed oil exhibited the highest toxicity against adult aphids, with an LC_50_ value of 1224.03 ppm, followed by *U. dioica* extract (1239.0 ppm), *H. muticus* extract (2297.24 ppm), and *S. chinensis* oil (3602.58 ppm). Sub-lethal effects of the tested botanical materials at LC_50_ concentrations were further investigated through biochemical analyses of total soluble proteins, total carbohydrates, and total lipids. Exposure to *C. tiglium* oil caused the greatest reduction in total protein and carbohydrate contents by 42.44% and 40.00%, respectively, whereas *U. dioica* extract reduced protein and carbohydrate levels by 36.95% and 26.66%, respectively, compared with the untreated control. In contrast, total lipid content significantly increased following treatment, reaching 47.61% and 33.33% above the control after exposure to *C. tiglium* oil and *U. dioica* extract, respectively. These findings demonstrate that the tested botanical extracts induce both toxic and metabolic disturbances in *A. gossypii* adults, suggesting their potential as eco-friendly alternatives for aphid management within integrated pest management programs.

**Supplementary Information:**

The online version contains supplementary material available at 10.1038/s41598-026-66584-0.

## Introduction

Citrus species, including navel orange, mandarin, and Valencia orange, represent one of the most important fruit crops in Egypt, due to its high nutritional and economic value^1–3^. However, citrus crops are frequently threatened by insect pests, which are among the most serious agricultural challenges in Egypt, causing substantial damage to orchards^4,5^. Worldwide, approximately twenty aphid species have been reported on citrus trees^6–8^, with the cotton aphid, *A. gossypii* Glover, recognized as a major pest due to its broad host range, colonizing over 600 plant species and transmitting more than 50 plant viruses^9–12^.

Traditional management of aphids relies heavily on synthetic insecticides, which has led to numerous challenges, including the development of resistant aphid strains, environmental contamination, and disruption of ecological balance^13^. Moreover, the potential carcinogenic, teratogenic, and mutagenic effects of conventional pesticides have raised considerable concern^14,15^. In response, recent research has focused on natural products as safer alternatives to synthetic chemicals. Plant-derived compounds are abundant sources of bioactive molecules, many of which are biodegradable, environmentally friendly, and effective even against insecticide-resistant populations^16–20^. Consequently, botanical pesticides are increasingly considered valuable tools in integrated pest management due to their low risk to both human health and the environment. For farmers in developing countries, plant extracts offer an efficient, safe, affordable, and easily accessible means of controlling insect pests^21^.

Beyond direct toxicity, plant extracts often exhibit a variety of sub-lethal effects on insect pests. These include growth retardation, reductions in fecundity and fertility, disruptions in molting, morphogenetic abnormalities and repellent activity have been reported in several insect species following exposure to botanical extracts^22,23^.

*Aphis gossypii* is considered one of the most destructive agricultural pests worldwide due to its extensive host range and its ability to cause severe direct and indirect crop losses. Heavy infestations can reduce plant vigor, induce leaf curling and chlorosis, and significantly decrease crop yield and quality. In citrus and vegetable crops, yield losses caused by aphid infestations may exceed 30–50% under severe infestation conditions. Moreover, *A. gossypii* acts as an efficient vector for more than 50 plant viruses, resulting in substantial economic losses in agricultural production systems worldwide^24^.

Therefore, the present study aims to investigate the toxicological effects and the biochemical alterations (total carbohydrates, proteins, and lipids) induced by selected botanical extracts and fixed oils against *Aphis gossypii*. It is hypothesized that exposure to these botanical materials may disrupt normal metabolic processes, leading to energy depletion and physiological stress in treated aphids. The present study was designed to evaluate both the toxicity and the biochemical disturbances induced by these botanical materials in adult aphids. Understanding these effects is essential for developing eco-friendly, sustainable, and effective strategies for aphid management, reducing reliance on synthetic pesticides, and mitigating their associated environmental and health risks.

## Materials and methods

### Rearing and breeding of *A. gossypii*

Adults of *A. gossypii* were collected from infested potato fields and transferred to potted plants maintained under controlled laboratory conditions at 25 ± 1 °C and 65 ± 5% relative humidity (RH). Colonies were allowed to establish on these plants for several generations. To sustain the aphid populations, plants were replaced every 5–7 days. All colonies were protected from external contamination by enclosing the infested plants in muslin cloth cages, which allowed adequate light penetration and ventilation while preventing access by predators and parasitoids.

### Phytochemical studies of tested samples

The present study evaluated four botanical materials: *C. tiglium* seed oil, *S. chinensis* seed oil, and ethanolic extracts of *U. dioica* and *H. muticus*. Plant materials were collected from Zagazig, Sharkia Governorate, Egypt. The collection was conducted according to local regulations, and no specific permits were required as the plants were collected from non-protected areas. Voucher specimens of the studied plant materials were prepared and deposited at the Herbarium of the Faculty of Science, Zagazig University, Egypt. The voucher specimen was assigned to the number ZU-2024-0121. The plant materials were taxonomically identified by Dr. [Mohamed Gad], Faculty of Science, Zagazig University.

### Extraction of fixed oils

Crushed seeds of *C. tiglium* and *S. chinensis* were ground into fine powder and soaked in petroleum ether (60–80 °C) at room temperature. The petroleum extracts were filtered using anhydrous sodium sulfate and stored in dark brown vials at 4 °C until further analysis.

### Determination of fatty acids

Free fatty acids were extracted from the crude oils according to the A.O.A.C^25^. method. The extracted fatty acids were converted into their corresponding methyl esters (FAMEs) and analyzed using gas–liquid chromatography (GLC) equipped with a flame ionization detector (FID), following the method described by Aren et al.^26^. Chromatographic separation was performed using a capillary column under programmed temperature conditions. Nitrogen was used as the carrier gas at a constant flow rate of 1 mL min^−1^. The injector and detector temperatures were maintained at 250 °C and 280 °C, respectively. The oven temperature was initially set at 180 °C and gradually increased to 240 °C at a rate of 4 °C min^−1^. Fatty acid components were identified by comparing their retention times with those of authentic standard fatty acid methyl esters. All analyses were carried out at the Food Technology Research Institute, Egypt.

### Isolation and identification of unsaponifiable fractions

Unsaponifiable compounds were analyzed using gas–liquid chromatography (GLC) according to the method described by Ramadan and Mörsel^27^. The analysis was carried out at the National Research Center, Cairo, Egypt, using a gas chromatograph equipped with a flame ionization detector (FID) and a capillary column suitable for hydrocarbon and sterol separation. Nitrogen was used as the carrier gas at a constant flow rate of 1 mL min^−1^. The injector and detector temperatures were maintained at 250 °C and 300 °C, respectively. The oven temperature program was initiated at 150 °C, gradually increased to 280 °C at a rate of 5 °C min^−1^, and held at the final temperature for 15 min. Identification of unsaponifiable compounds was achieved by comparing retention times with those of authentic reference standards and published data.

### Ethanolic extract preparation and analysis

Aerial parts of *U. dioica* and *H. muticus* were dried, ground to a fine powder (< 300 µm), and extracted with ethanol. Thin Layer Chromatography (TLC) was used to fractionate the crude extracts, using silica gel plates and appropriate solvent systems: ethyl acetate:methanol (4:1) for *U. dioica* and ethanol:acetate:water (5:2:1) for *H. muticus*. Visualization was achieved with iodine vapor, and Rf values were calculated following El-Sadany et al.^28^. Selected fractions were purified by column chromatography, extracted with acetone, and concentrated under reduced pressure.

### Identification of fractional compounds

Purified fractions were characterized using UV and IR absorption spectra (Perkin Elmer 1650 FT-IR) and GC/MS analysis (Shimadzu GC/MS-QP-1000-Ex, Japan) to determine empirical formulae and chemical structures^29^.

### Toxicological studies

Stock solutions of *Croton tiglium* and *Simmondsia chinensis* seed oils were prepared by emulsifying the oils in 0.1% Tween 80 as an emulsifying agent, followed by dilution with distilled water to obtain the required concentrations (625, 1250, 2500, 5000, 10,000, and 20,000 ppm). Ethanolic extracts of *Urtica dioica* and *Hyoscyamus muticus* were similarly prepared at the same concentration range using distilled water containing 0.1% Tween 80. A control treatment containing distilled water and Tween 80 only was included in all experiments. Citrus leaf discs were dipped individually in each prepared concentration for 10 s, then allowed to air-dry for approximately 30 min at room temperature before being transferred into three Petri dishes. Each concentration was tested in three biological replicates, with each replicate consisting of 30 adult aphids placed in a separate Petri dish (25 ± 1 °C and 65 ± 5% RH). Mortality was recorded after 48 h of exposure.

Mortality percentages were corrected according to Abbott’s formula^30^. Median lethal concentration (LC_50_) values and confidence limits were estimated using probit analysis following Finney^31^. Toxicity index values were calculated according to Sun^32^ using the following equation:

$${\text{Toxicity index}} = \left( {{\mathrm{LC}}_{{{5}0}} {\text{of the most effective compound}}/{\mathrm{LC}}_{{{5}0}} {\text{of the tested compound}}} \right) \times {1}00$$

Relative potency was calculated according to Zidan and Abdel-Maged^33^ as follows:

$${\text{Relative potency}} = {\mathrm{LC}}_{{{5}0}} {\text{of the least effective compound}}/{\mathrm{LC}}_{{{5}0}} {\text{of the tested compound}}$$

### Biochemical studies

Infested plants were divided into five groups: four treatment groups and one control group. Each treatment group was exposed to the LC_50_ concentration of one of the tested botanical materials, namely *Croton tiglium*, *Simmondsia chinensis*, *Urtica dioica*, or *Hyoscyamus muticus*, while the fifth group was treated with distilled water as a control. Adult aphids (1 g per treatment) were collected 48 h post-treatment, homogenized in distilled water, and centrifuged at 5000 rpm for 10 min at 5 °C. The resulting supernatant was used for biochemical analyses.

Total soluble proteins were determined according to Bradford^34^, total carbohydrates were estimated following Crompton and Birt^35^, and total lipids were quantified according to Knight et al.^36^. All biochemical determinations were performed in three biological replicates, with each assay run in triplicate technical measurements to ensure accuracy and reproducibility.

Calculation of change percentage of total protein, carbohydrate and lipid were calculated from

$$\begin{aligned} {\mathrm{C}}\% \, \left( {{\text{Change }}\% } \right) & = \frac{{{\text{Treatment }} - {\text{ Control}}}}{{{\mathrm{Control}}}} \times 100 \\ & \quad {\text{and the concentration is expressedas }}\left( {\upmu {\mathrm{g}}/{\mathrm{mg}}} \right) \\ \end{aligned}$$

%; Percent decrease or increase from the control.

(+) or (−) refers to the percentage increase or decrease from the control.

*; Significant (*P* < 0.05).

**; Highly significant (*P* < 0.001).

### Statistical analysis

Biochemical data, including total proteins, carbohydrates, and lipids, were subjected to one-way analysis of variance (ANOVA). Prior to analysis, data were tested for normality and homogeneity of variance. Mean comparisons among treatments were performed using Tukey’s HSD post-hoc test at *P* ≤ 0.05. LC_50_ values, confidence intervals, and slope values were estimated by probit analysis according to Finney^31^.

**Fig. 1:**
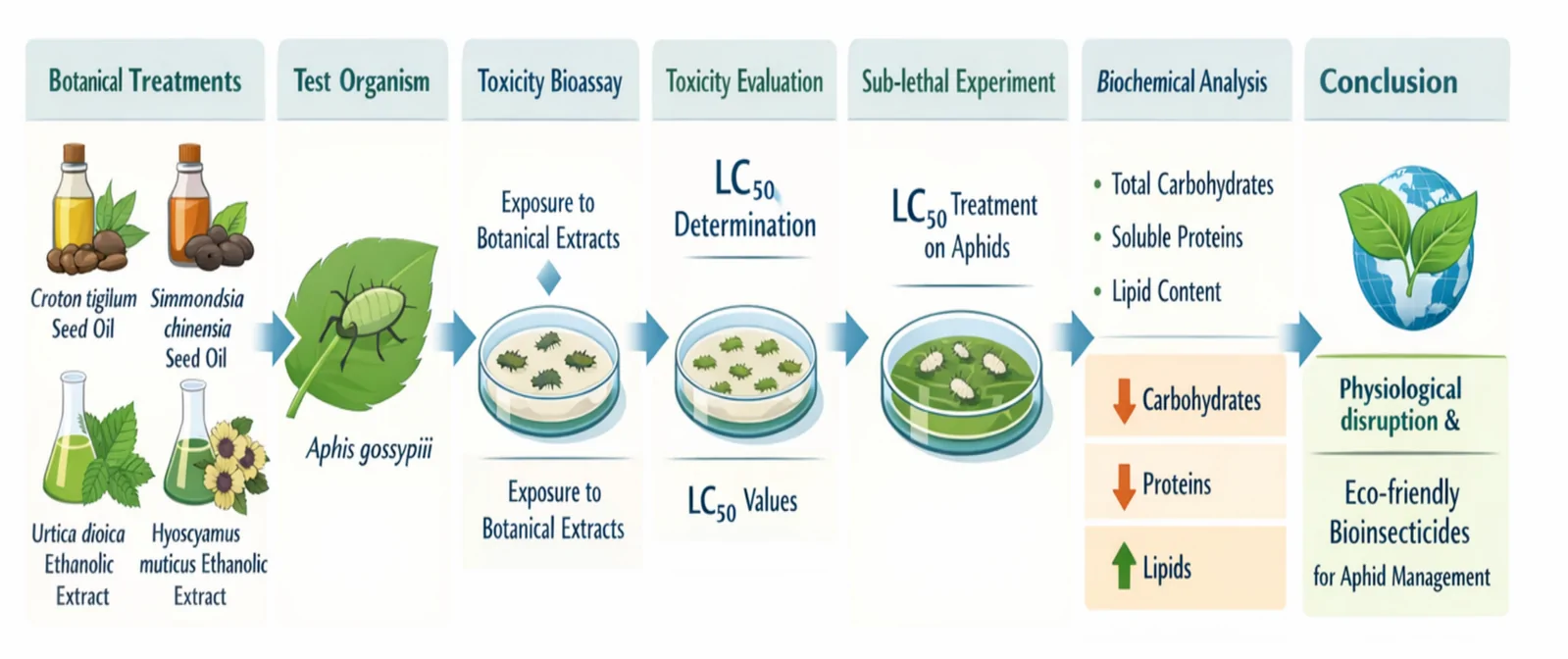
Workflow of the toxicological and biochemical assessment of botanical extracts on *Aphis gossypii.*

To visually summarize the experimental design, a workflow diagram (Fig. 1) was created, illustrating the study on the toxicological and biochemical effects of botanical extracts on *Aphis gossypii*. The diagram outlines the adult aphid as the test subject, the four tested botanical samples (*Croton tiglium* seed oil, *Simmondsia chinensis* seed oil, *Urtica dioica* ethanolic extract, and *Hyoscyamus muticus* ethanolic extract), the experimental procedures including phytochemical analysis, toxicological assessment, and biochemical assays, and the resulting effects such as toxicity ranking and sub-lethal biochemical changes.

## Result and discussion

### Phytochemical studies

#### Identification of free fatty acids (fixed oils)

The analytical data obtained from GLC spectrum of methylated fatty acid fraction shown in (Figs. S1 and S2) and presented in (Table 1), showed that there are seven fatty acids of *Corton tiglum* seeds oil, three of them are saturated; Myristic, Palmetic and Methyl Arachidanate (the major), all are with 45.48%. Four unsaturated fatty acids were detected namely Oleic (the major), Linoleic, Methyl eicosanoate and Eicosadienoic acid. While there are eleven fatty acids of *Simmondsia chinesis* seeds oil, four of them are saturated; Myristic, Palmetic (the major), Stearic and Methyl Arachidanate, all are with 12.77%. Seven unsaturated fatty acids; Myristoleic, Palmetoleic, Oleic (the major), Linoleic, Linolenic, Methyl eicosanoate and Arachidonic acid, these results were supported by^37^ who proved that the major components of *C. tiglium* seeds oil was oleic acid, palmitic acid and hexadeconic acid. In the contrast, Wael et al.^38^ proved that 11,14-Octadecadienoic acid and Hexadecanoic acid were the major unsaturated and saturated fatty acid, respectvely in *C. tiglium* seeds oil, Al-Qizwini et al.^39^ reported that α-linolenic acid was in higher % in *S. chinensis* oil, and Al-Ghamdi et al.^40^ showed that Behenic acid methyl ester and Cis-13, 16 Docosadienoic acid methyl ester were the highest in % in *S. chinensis* oil.

**Table 1:** Free fatty acids and their relative percentage in *C. tiglium* and *S. chinensis* oils analyzed by gas liquid chromatography (GLC).

Fatty acid		*C. tiglium*		*S. chinensis*	
Name	Carbon n	Ret. time	%	Ret. time	%
Myristic acid	C14(0)	11.17	3.63	12.61	1.07
Myristoleic acid	C14(1)	–	–	13.36	0.38
Palmetic acid	C16(0)	12.62	15.49	14.18	10.49
Palmetoleic acid	C16(1)	–	–	14.76	0.29
Stearic acid	C18(0)	–	–	15.06	0.36
Oleic acid	C18(1)	13.98	29.43	15.85	70.72
Linoleic acid	C18(2)	15.27	18.53	17.19	10.27
Linolenic acid	C18(3)	–	–	17.69	4.87
Mythyl arachidanate acid	C20(0)	17.29	26.36	18.71	0.85
Methyl eicosanoate acid	C20(1)	17.48	4.41	18.9	0.45
Eicosadie acid	C20(2)	17.7	2.13	–	–
Arachidonic acid	C20(4)	–	–	19.5	0.16
Total saturated fatty acid			45.28		12.77
Total unsaturated fatty acid			54.72		87.23

**Fig. 2:**
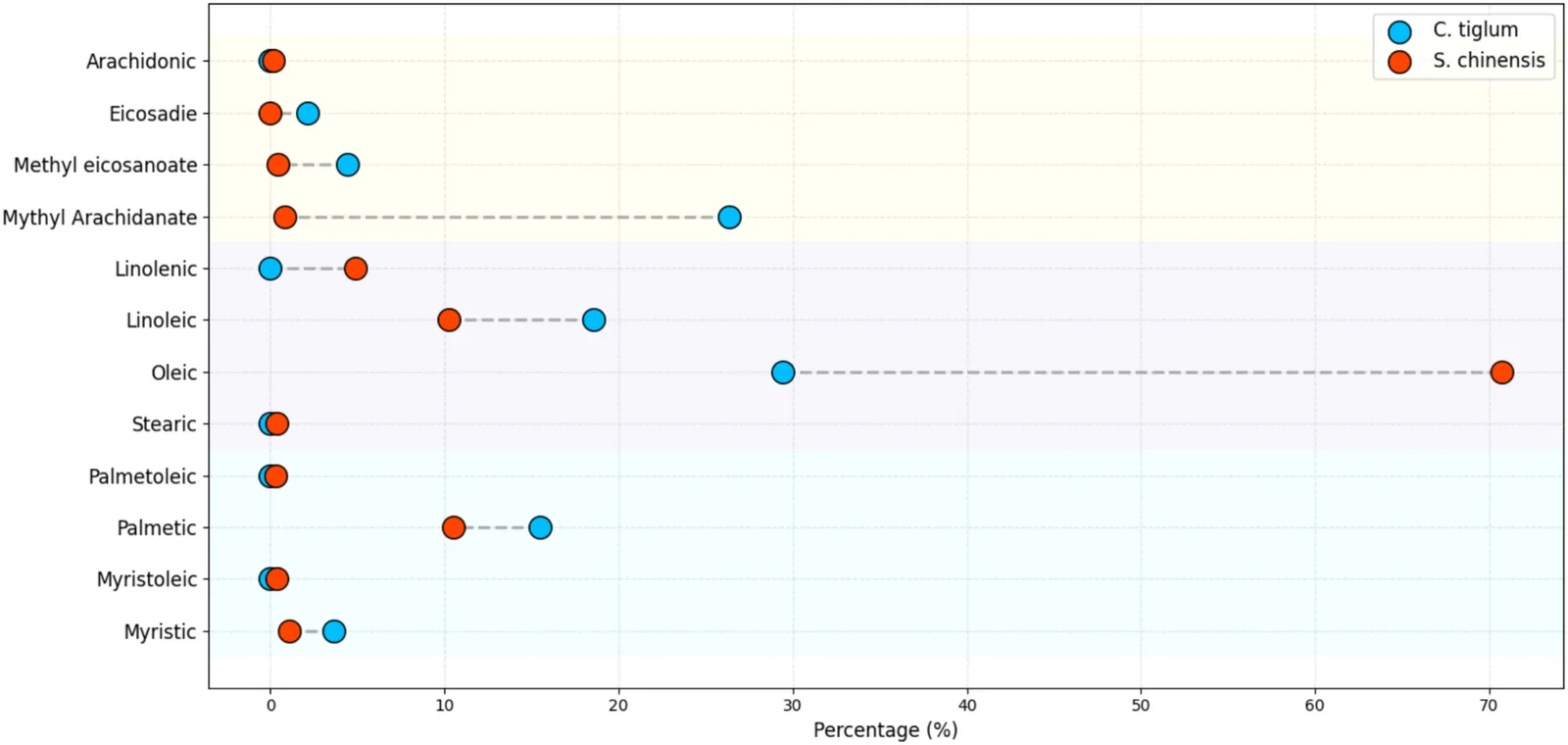
Comparative dot plot of fatty acid composition in *C. tiglium* and *S. chinensis* oils with highlighted functional groups.

The dot plot Fig. 2 illustrates the comparative composition of fatty acids in *C. tiglium* and *S. chinensis* seed oils. Each fatty acid is represented by two points connected by dashed lines, indicating the variation in relative percentage between the two oils. Background shading highlights different groups of fatty acids, such as saturated, monounsaturated, and polyunsaturated types, providing a clear visual distinction. The visual connections allow rapid identification of fatty acids with large differences, facilitating an intuitive understanding of the comparative biochemical profiles.

#### Identification of unsaponifiable component

GLC analysis of unsaponifable component of *C. tiglium* and *S. chinensis* seeds are shown in (Figs. S3 and S4) and data in (Table 2) demonstrated that *C. tiglium* seeds oil contain 13 compounds; 9 hydrocarbons where Tetracosane was the major with relative percentage 15.59% and 4 sterols where B- Stitosterol was the major with relative percentage 17.75%. While *S. chinensis* seed oil contain 9 hydrocarbons; where Tricosane was the major components with relative percentage 49.55%, while contain two sterol components Stigmasterol (2%) and B-Sisterol (1%). Our results in agreement with Tada et al.^41^ and El-Mallah and El-Shami^42^ who proved that Sterols and stanols are mainly represented by; campesterol, stigmasterol, sitosterol, cholesterol and avenasterol in jojoba seeds oil.

**Table 2:** Sterols and hydrocarbon and their relative percentage in *C. tiglium* and *S. chinensis* oils analyzed by gas liquid chromatography (GLC).

	Name	Formula	Structural formula	*C. tiglium*		*S. chinensis*	
				Ret. time	%	Ret. time	%
Hydrocarbon	Pentadecane	C_15_H_32_		11.69	1.65	11.78	0.2
	Hexadecane	C_16_H_34_		12.29	1.25	12.23	0.01
	Heptadecane	C_17_H_36_		13.84	1.79	13.57	0,009
	Octadecane	C_18_H_38_		14.95	3.36	–	–
	Nonadecane	C_19_H_40_		16.45	3.1	16.25	0.15
	Eicosane	C_20_H_42_		17.36	1.93	17.79	0.26
	Henicosane	C_21_H_44_		18.51	2.64	18.53	1,66
	Docosane	C_22_H_46_		19.58	2.67	19.73	0.53
	Tricosane	C_23_H_48_		21.61	3.65	21.96	49.55
	Tetracosane	C_24_ H_50_		25.6	15.59	24.57	31.25
	Hexcosane	C_26_H		26.74	1.57	26.43	3.12
	Octacosane	C_28_H_56_		28.31	6.32	28.39	9.96
Sterols	Cholesterol	C_27_H_46_O		29.26	4.04	–	–
	Campasterol	C_28_H_48_O		30.36	5.7	–	–
	Stigmasterol	C_29_H_48_O		32.07	17.48	33.07	2.13
	B- Sitosterol	C_29_H_50_O		34.53	17.75	34.29	1.77

**Fig. 3:**
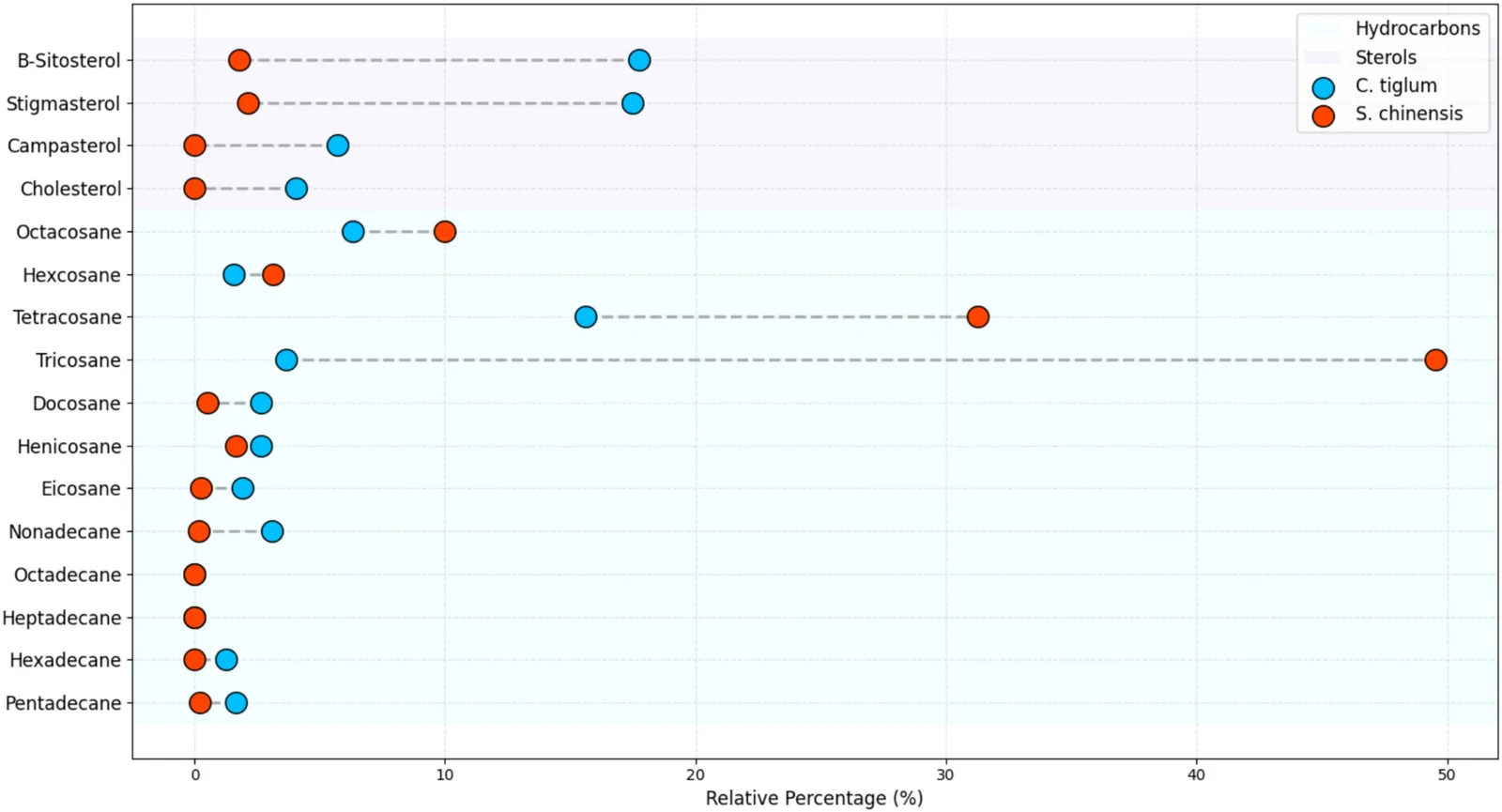
Comparative dot plot of hydrocarbon and sterol profiles in *Croton tiglium* and *Simmondsia chinensis* seed oils.

Comparative visualization Fig. 3 of the unsaponifiable fractions revealed marked differences in the distribution of hydrocarbons and sterols between *C. tiglium* and *S. chinensis* seed oils. Several hydrocarbons, such as tricosane and tetracosane, showed higher relative abundance in *S. chinensis*, whereas *C. tiglium* oil exhibited greater proportions of specific sterols, particularly β-sitosterol and stigmasterol. The dot plot representation allowed clear identification of compounds with pronounced variation, emphasizing the distinct chemical fingerprints of the two botanical oils.

### Identification of miscellanous compounds

#### Identification of empirical and structural formulae of major compound of ethanolic extract of Utrica; Urtica dioica

**Fig. 4:**
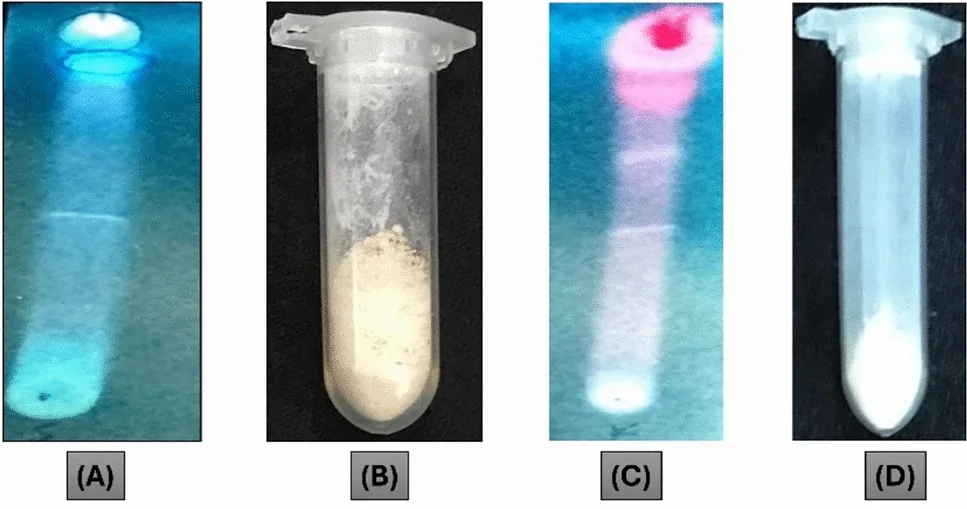
Miscellaneous compounds of *U. dioica* and *H. muticus* (**A**) Ethanolic extract of *Urtica dioica* in developing system (Ethanol: Acetate: water 5:2:1), (**B**) The purified compound of *Urtica dioica* (**C**) Ethanolic extract of *Hyoscyamus muticus* in developing system (Ethyl acetate: Methanol 4:1), (**D**) The purified compound of *Hyoscyamus muticus*.

The ethanolic extract of *Urtica dioica* produced six distinct bands upon preparative thin-layer chromatography (PTLC) using a solvent system composed of ethyl acetate and methanol (4:1, v/v) on 20 × 20 cm plates. Among these fractions, only the predominant band with an Rf value of 0.80 was selected for purification and subsequent identification. The isolated compound appeared as a fine, pale green powder, as illustrated in Fig. 4.

**Fig. 5:**
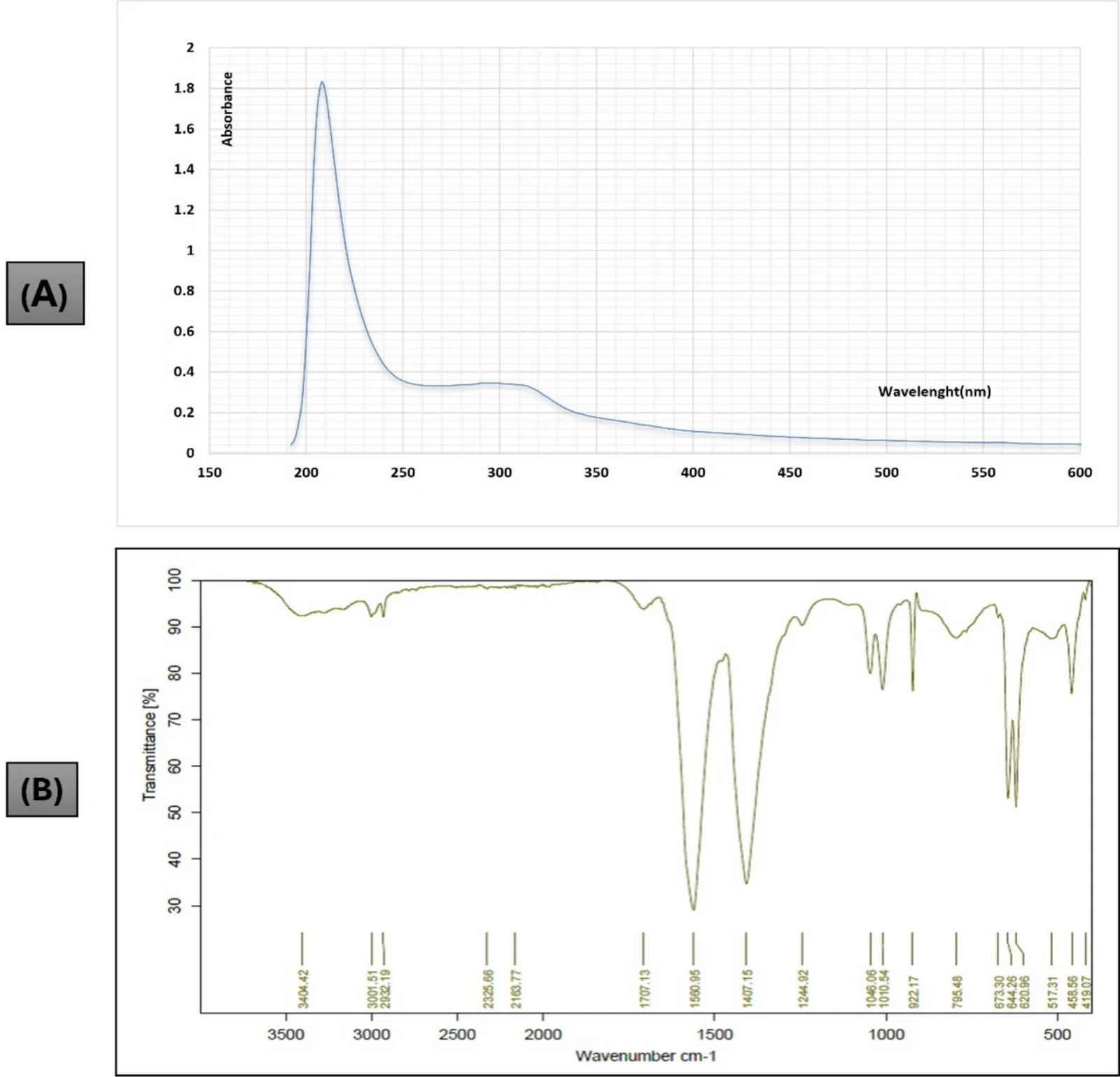
(**a**) U. V. Spectrum of Epicatechin, isolated from ethanolic extract of *Urtica dioica*. (**b**) I.R. Spectrum of Epicatechin, isolated from ethanolic extract of *Urtica dioica*.

The ultraviolet (UV) absorption spectrum of the isolated compound (Fig. 5a) exhibited a strong absorption band at 210 nm, which is characteristic of a heterocyclic molecular framework. In addition, a weaker absorption band was observed at 298 nm, accompanied by a broad shoulder around 300 nm. These spectral features are commonly associated with the presence of aromatic ring systems and conjugated π–electron structures, indicating a complex molecular arrangement with aromatic functionality.

Infrared (IR) spectroscopic analysis (Fig. 5b) further supported the structural characterization of the compound. The spectrum displayed a broad absorption band at 3404 cm^−1^, suggesting the presence of conjugated hydroxyl (O–H) groups and/or amine (N–H) functionalities. Absorption at 3011 cm^−1^ was attributed to aromatic C–H stretching vibrations, whereas the band at 2932 cm^−1^ corresponded to aliphatic C–H stretching modes. A bending vibration observed at 1707 cm^−1^ indicated the possible presence of C–O–N linkages within the molecular structure. Furthermore, a distinct absorption band at 1560 cm^−1^ was assigned to aromatic C=C stretching vibrations, while peaks at 1504 and 1407 cm^−1^ suggested N–H bending or carbon–carbon skeletal vibrations. The presence of a band at 1244 cm^−1^ was indicative of C–N stretching, whereas absorption bands at 1046 and 1010 cm^−1^ confirmed C–O functional groups. Additional bands appearing in the range of 673–620 cm^−1^ further supported the existence of N–H and C–N bonds. Collectively, these spectral features reveal the presence of multiple functional groups, including N–H, C–H, C=C, C=O, C–N, and C–O–C linkages, in conjunction with aromatic ring systems. Such a combination of structural elements strongly suggests that the compound belongs to the alkaloid class, most likely within the oxindole category, in agreement with the findings reported by^43,44^.

**Fig. 6:**
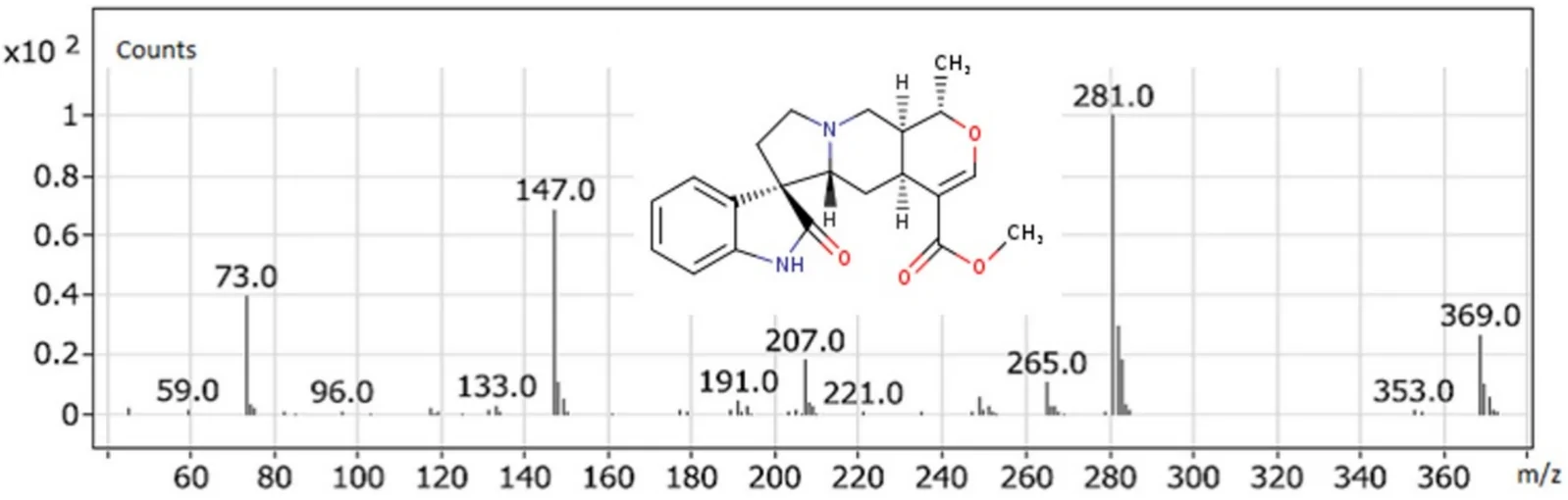
Mass spectrum of Epicatechin, isolated from ethanolic extract of *Urtica dioica*.

Mass spectrometric analysis of the compound (Fig. 6) provided additional confirmation of its molecular identity. The spectrum exhibited a prominent molecular ion peak (M^+^) at m/z 369, along with an isotopic peak (M^+^ + 1) at m/z 370, corresponding to the intact molecular structure. Moreover, several high-intensity fragment ions were detected at m/z 281, 147, and 73, which represent characteristic fragmentation patterns of the compound. Comparison of the obtained mass spectral data with reference spectra from the European MassBank database revealed a strong similarity to Uncarine derivatives. This identification is further supported by previously reported mass spectrometric data^45–47^, thereby confirming the proposed structural assignment.

All obtained data refer that the isolated compound which apparently having the following formula:- Chemical name: 19 α-Methyl-2-oxoformosanan-16-carboxylic acid methyl ester (Uncarine F). Emprical formula: C21H24N2O4.

#### Identification of the empirical and structural formulae of the ethanolic extract fraction of Egyptian henbane (*Hyoscyamus muticus*)

Preparative thin-layer chromatography (PTLC) of the ethanolic extract of Egyptian henbane (*Hyoscyamus muticus*) resulted in the separation of four major fractions using a solvent system composed of ethanol, ethyl acetate, and water in a ratio of 5:2:1 (v/v/v). Among these fractions, only the predominant band, exhibiting an Rf value of 0.76, was selected for further purification and structural identification. The isolated compound was obtained as a fine powder with a characteristic yellowish coloration, as illustrated in Plate II.

**Fig. 7:**
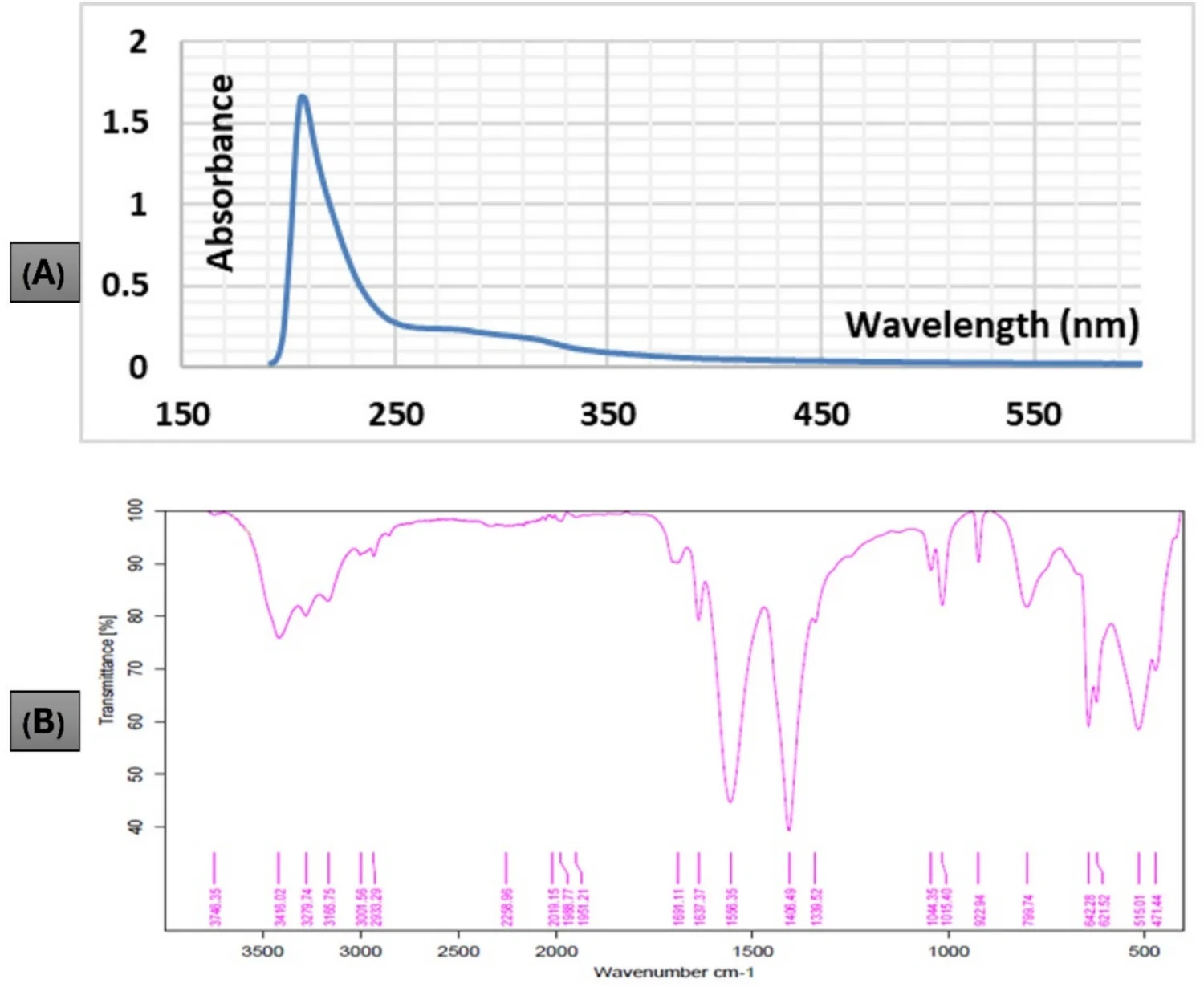
Mass spectrum, p-Cresol, 2,2'-methylenebis(6-tert-butyl) isolated from ethanolic extract of *Hyoscyamus muticus.*

Ultraviolet (UV) spectroscopic analysis of the purified compound (Fig. 7a) revealed a strong absorption band at 208 nm, which is indicative of π–π* electronic transitions commonly associated with aromatic or conjugated molecular systems. This absorption feature suggests the presence of a chromophoric group within the molecular structure.

Infrared (IR) spectral analysis (Fig. 7b) provided further insights into the functional groups present in the compound. Broad stretching absorption bands observed at 3416, 3279, and 3165 cm^−1^, along with bands at 1044 and 1015 cm^−1^, indicated the presence of multiple hydroxyl (–OH) groups. Strong absorption peaks at 3001 and 2924 cm^−1^, as well as a band at 1406 cm^−1^, were attributed to aliphatic C–H stretching and bending vibrations. Additionally, absorption bands at 1691 and 1639 cm^−1^, together with a peak at 1339 cm^−1^, suggested the presence of carbon–carbon double bonds (C=C). The distinct absorption peak at 1556 cm^−1^ was characteristic of a carbonyl (C=O) group, likely corresponding to a ketone functional moiety. Collectively, these spectral features are consistent with a phenolic or triterpenoid-type structure.

**Fig. 8:**
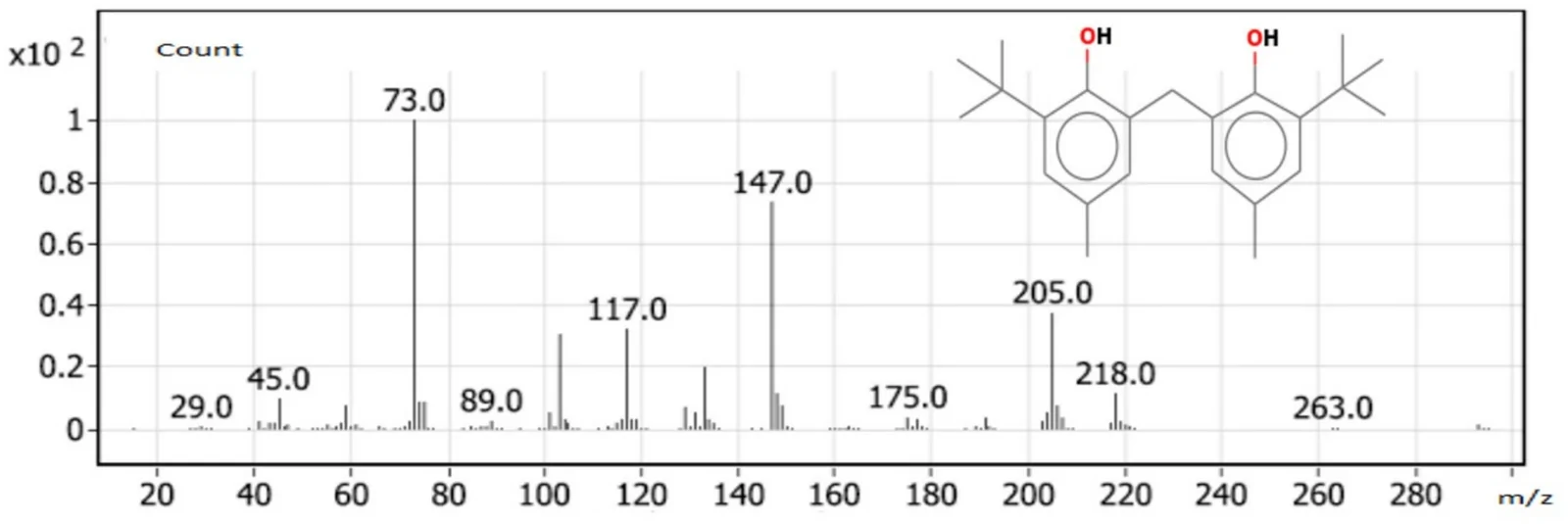
(**a**) U.V.spectrum, p-Cresol, 2,2'-methylenebis(6-tert-butyl) isolated from ethanolic extract of *Hyoscyamus muticus* (**b**) IR spectrum of p-Cresol, 2,2'-methylenebis(6-tert-butyl) isolated from ethanolic extract of *Hyoscyamus muticus.*.

Mass spectrometric analysis of the compound (Fig. 8) revealed a molecular ion peak (M^+^) at m/z 263 with a relative intensity of 5%, along with several prominent fragment ions at m/z 218 (15%), 205 (35%), 175 (20%), 147 (86%), 89 (35%), and 73 (100%). These fragmentation patterns correspond closely to those reported for p-Cresol, 2,2′-methylenebis(6-tert-butyl). The identification was further confirmed through comparison with published mass spectral data, in agreement with the findings of Eman et al.^48^.

### Toxicological studies

#### Toxic effects against adult *Aphis gossypii* under laboratory conditions

Under controlled laboratory conditions, the toxicological efficacy of two plant-derived fixed oils and two ethanolic extracts was evaluated against the adult stage of *Aphis gossypii*. The assessment aimed to determine the median lethal concentration (LC_50_), defined as the concentration required to cause 50% mortality of the tested aphid population.

#### Susceptibility of adult *Aphis gossypii* to the tested fixed oils

**Fig. 9:**
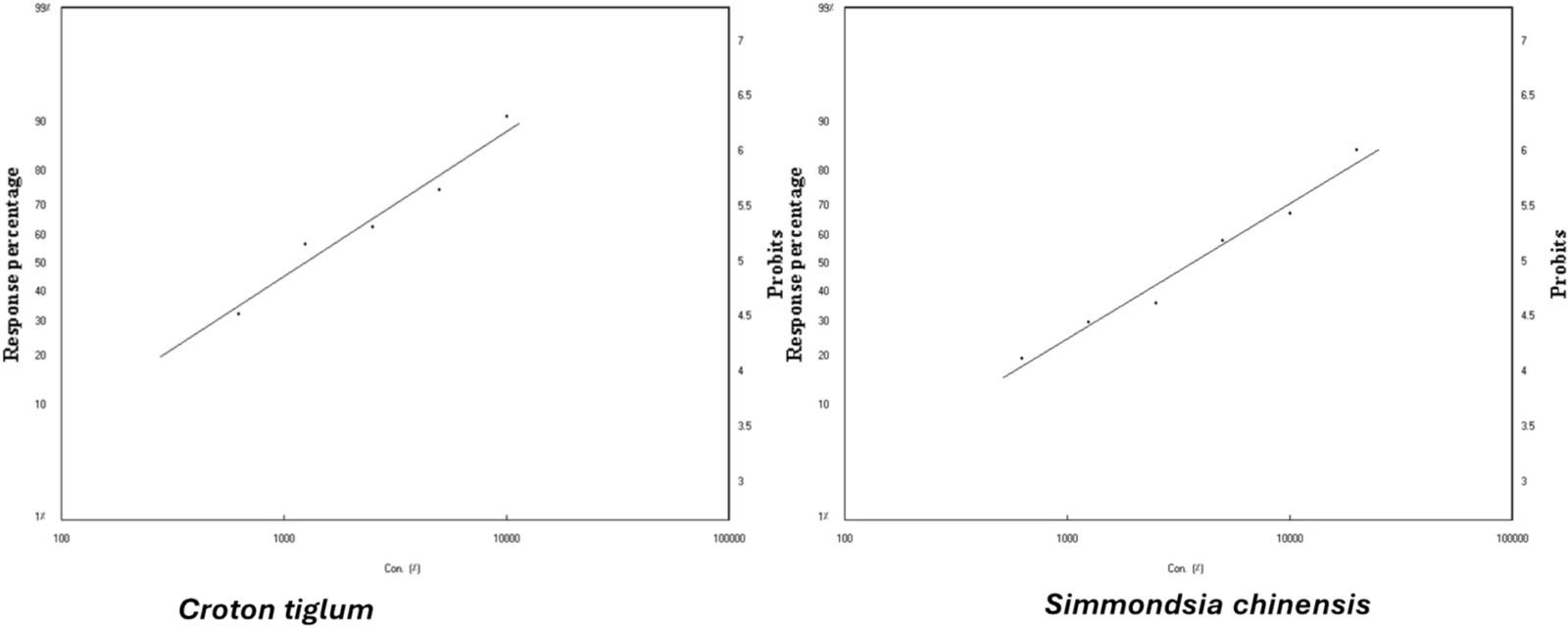
Log. Conc- Mortality regression line of the plant fixed oils against adult *A. gossypii*.

The susceptibility of adult *A. gossypii* to the two fixed oils is presented in Table 3 and illustrated in Fig. 9. The results demonstrated clear differences in toxicity between the tested oils. The LC_50_ values were estimated at 3602.8 ppm for *Simmondsia chinensis* (jojoba) oil and 1244.33 ppm for *Croton tiglium* oil. Based on these values, *C. tiglium* oil exhibited markedly higher toxicity against adult aphids compared with *S. chinensis* oil.

**Table 3:** Toxicity of *Croton tiglum* and *Simmondsia chinensis* fixed oils against adult of *Aphis gossypii*.

Tested plants	Conc. (ppm)	Mortality%	LC_50_ ppm	Confidence limits of LC_50_		Toxicity index %	Relative potency	Slope
				Lower	Upper			
*Croton tiglum*	20,000	100 ± 00	1224.03	925.40	1526.87	100	2.92	1.32
	10,000	91.00 ± 2.65						
	5000	75.00 ± 4.58						
	2500	63.33 ± 5.03						
	1250	57.27 ± 4.82						
	625	32.63 ± 4.72						
*Simmondsia chinensis*	20,000	85.00 ± 4.58	3602.58	2936.75	4423.45	33.97	1.00	1.22
	10,000	68.17 ± 5.97						
	5000	58.57 ± 5.70						
	2500	36.17 ± 5.43						
	1250	29.90 ± 4.82						
	625	19.63 ± 4.46

The toxicity index of *S. chinensis* oil was calculated to be 34.7% relative to the most effective treatment, which was assigned a toxicity index of 100%. Furthermore, the relative potency analysis revealed that *C. tiglium* oil was approximately 2.92-fold more toxic than *S. chinensis* oil. These findings clearly indicate that *C. tiglium* fixed oil possesses superior insecticidal activity against adult *A. gossypii* when evaluated in terms of LC_50_ values, toxicity index, and relative potency.

The higher toxicity exhibited by *C. tiglium* oil in comparison with *S. chinensis* oil may be attributed, at least in part, to its substantially greater total sterol content, which was recorded as 44.97% for *C. tiglium* and only 3.9% for *S. chinensis* (Table 3). Sterols are known to play critical roles in insect physiology. In this context, Canavoso et al.^49^ reported that cholesterol constitutes the principal sterol in insects, where it functions as an essential structural component of cell membranes and serves as a precursor for ecdysteroids, the hormones responsible for regulating insect moulting and development. Notably, even when adequate amounts of alternative sterols such as sitosterol are available, insect development may still be adversely affected. For instance, sitosterol was shown to impede normal growth and development in the desert locust *Schistocerca americana*. Conversely, tobacco hornworm larvae exhibited only mild toxic effects when fed diets enriched with cholesterol; however, exposure to equivalent concentrations of sitosterol resulted in pronounced toxicity in the same insect species. These findings suggest that the type and composition of sterols present can significantly influence insect survival and development.

Moreover, the observed toxicity of the saponifiable fraction may be associated with the presence of unsaturated long-chain fatty acids, including oleic, linoleic, and linolenic acids, as well as the non-polar long-chain hydrocarbons constituting the unsaponifiable fraction. These components were detected at relatively high proportions in both *C. tiglium* and *S. chinensis* oils. Unsaturated fatty acids are known to readily penetrate insect cell membranes, where they may disrupt membrane integrity and interfere with essential physiological processes. Additionally, their accumulation within cells can lead to metabolic imbalance and cellular dysfunction. Such mechanisms have been previously documented by^50–54^ supporting the proposed mode of action of these botanical oils against insect pests.

### Susceptibility of adult *Aphis gossypii* to the tested ethanolic extracts

**Fig. 10:**
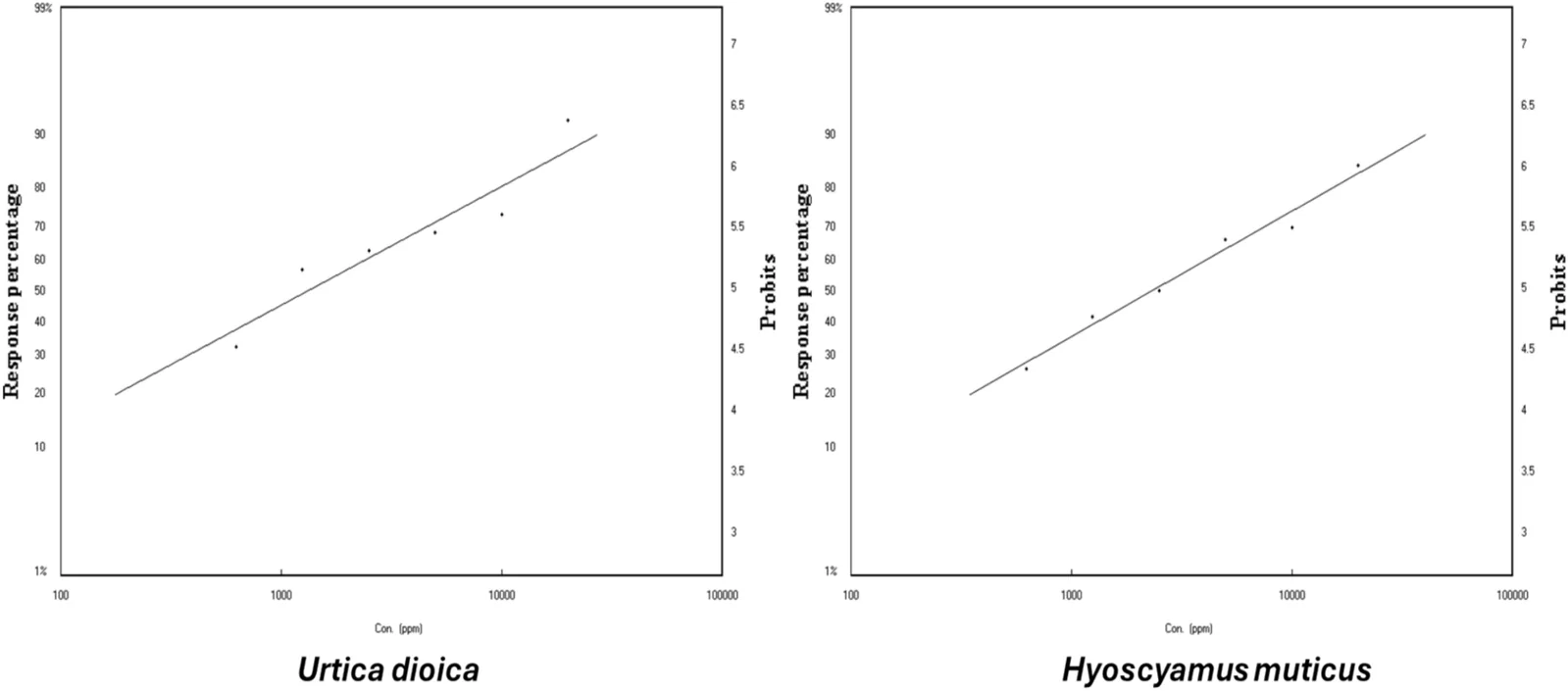
Log. Conc- Mortality regression line of the plant ethanolic extracts against adult *A. gossypii*.

The toxicological response of adult *Aphis gossypii* to the ethanolic extracts of *Urtica dioica* and *Hyoscyamus muticus* is presented in Table 4 and illustrated in Fig. 10. The results indicate that both botanical extracts exhibited appreciable insecticidal activity; however, marked differences in efficacy were observed between the two treatments. The median lethal concentration (LC_50_) values were estimated at 1239.0 ppm for *U. dioica* and 2297.24 ppm for *H. muticus*, demonstrating that *U. dioica* extract was considerably more toxic to adult aphids.

**Table 4:** Toxicity of *Urtica dioica* and *Hyoscyamus muticus* ethanolic extracts against adult of *Aphis gossypii*.

Tested plants	Con (ppm)	Mortality%	LC50 ppm	Confidence limits of LC50		Toxicity index %	Relative potency	Slope
				Lower	Upper			
*Urtica dioica*	20,000	92.00 ± 3.61	1239.0	836.39	1664.94	100	1.85	0.9441
	10,000	73.67 ± 5.08						
	5000	68.50 ± 5.53						
	2500	63.70 ± 5.45						
	1250	57.03 ± 5.13						
	625	34.77 ± 4.87						
*Hyoscyamus muticus*	20,000	85.00 ± 4.58	2297.24	1753.31	2915.94	53.93	1.00	1.028
	10,000	70.00 ± 5.57						
	5000	66.57 ± 5.90						
	2500	50.47 ± 5.66						
	1250	41.77 ± 5.22						
	625	26.47 ± 4.86

Based on LC_50_ values, the toxicity index of *U. dioica* extract was assigned as 100%, whereas that of *H. muticus* extract was calculated to be 53.00%. Furthermore, the relative potency analysis revealed that the ethanolic extract of *U. dioica* was approximately 1.8-fold more toxic than that of *H. muticus*. These findings clearly indicate the superior insecticidal efficiency of *U. dioica* extract against adult *A. gossypii* under laboratory conditions.

Thus, *U*. *dioica* extract is the most potent extract and has highest insecticidal activity than *H. muticus* against *Aphis gossypii* adult stage.

### Biochemical studies

#### Biochemical effects on the adult stage of *Aphis gossypii*

Newly emerged adults of *Aphis gossypii* were exposed to the median lethal concentration (LC_50_) of the tested fixed plant oils (*Croton tiglium* and *Simmondsia chinensis*) and ethanolic plant extracts (*Urtica dioica* and *Hyoscyamus muticus*) for a period of 48 h under laboratory conditions. Following treatment, the total soluble protein, total lipid, and total carbohydrate contents were quantitatively determined to assess the biochemical alterations induced by these botanical compounds.

#### Total soluble protein content

**Fig. 11:**
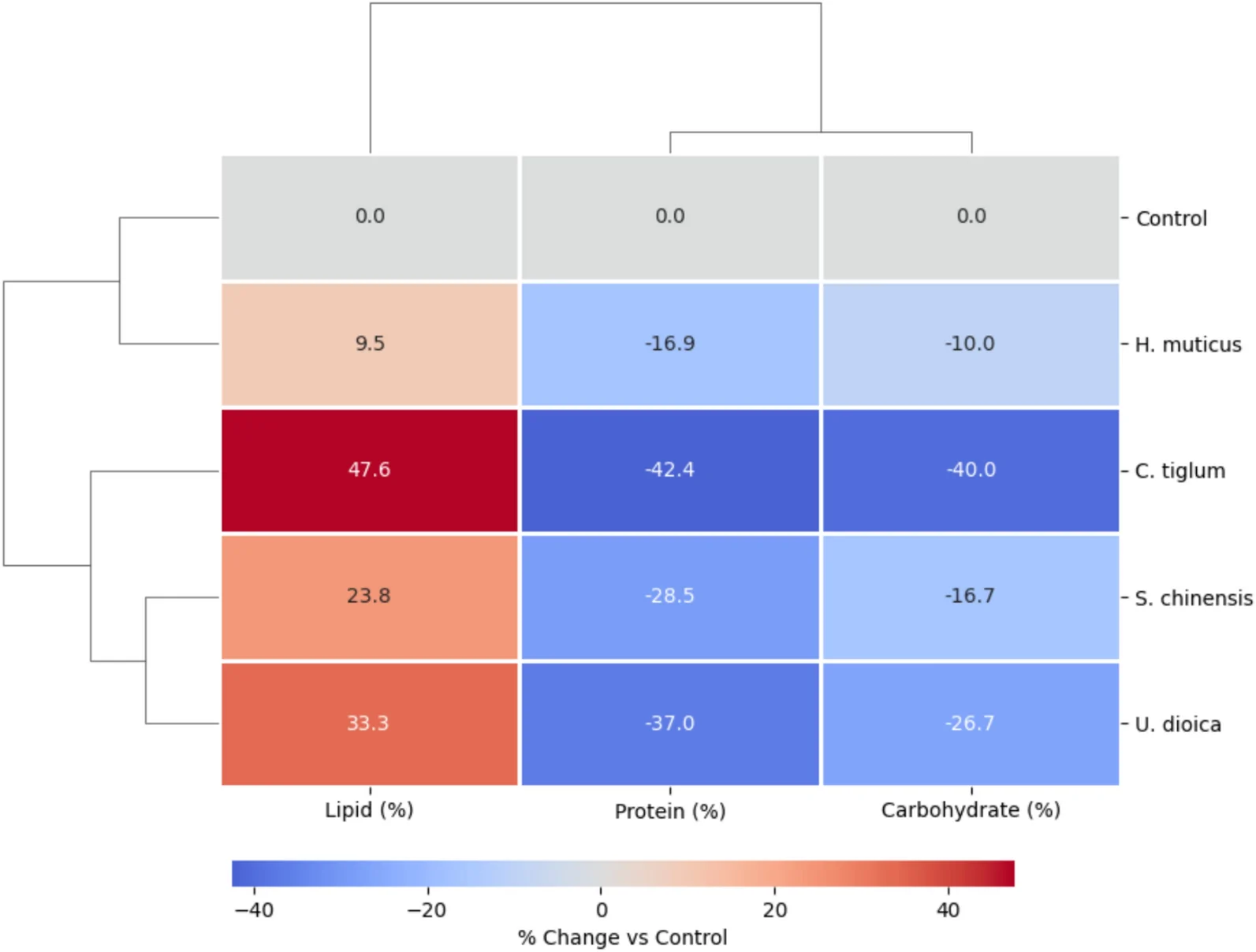
Heatmap illustrating the percentage changes in total protein, total lipid, and total carbohydrate contents of adult *Aphis gossypii* treated with LC_50_ concentrations of fixed oils (*C. tiglium* and *S. chinensis*) and ethanolic extracts (*U. dioica* and *H. muticus*).

The data presented in Table 5 and illustrated in Fig. 11 indicate that treatment of adult *A. gossypii* with the LC_50_ concentrations of the two fixed oils (*C. tiglium* and *S. chinensis*) and the two ethanolic extracts (*U. dioica* and *H. muticus*) resulted in a significant reduction in total soluble protein content compared with the control. Among the tested treatments, *C. tiglium* oil and *U. dioica* extract exhibited the most pronounced effects, inducing protein reductions of 42.44% and 36.95%, respectively, relative to untreated aphids.

**Table 5:** Change of total protein, lipid and carbohydrate levels (µg/mg fresh body weight) in the adult of *A. gossypii* treated with the LC_50_ tested compounds for 48 h.

Treatment	Conc. LC_50_ Ppm	Total protein		Total lipid		Total carbohydrate	
		µg/mg	%	µg/mg	%	µg/mg	%
*C. tiglium*	1244.33	20.34 ± 1.08*	− 42.44	155.00 ± 3.16*	47.61	18.00 ± 0.85*	− 40.00
*S. chinensis*	3584.68	25.20 ± 1.46*	− 28.46	130.00 ± 2.60*	23.80	25.00 ± 1.58*	− 16.66
*U.dioica*	1224.11	22.28 ± 1.34*	− 36.95	140.00 ± 3.40*	33.33	22.00 ± 1.30*	− 26.66
*H. muticus*	2309.26	29.38 ± 0.60*	− 16.86	115.00 ± 2.00*	9.52	27.00 ± 0.70*	− 10.00
Control	–	35.34 ± 1.49	–	105.00 ± 1.41	**–**	30.00 ± 1.30	–

These findings are consistent with previous studies reporting that botanical extracts and plant-derived oils can markedly reduce protein levels in insects^55–59^. Protein depletion may reflect disruption of protein synthesis, enhanced proteolysis, or utilization of proteins as alternative energy sources under stress conditions.

In contrast, variable responses in protein content have been documented in other insect species. For instance, Askar et al.^56^ and Draz et al.^59^ reported mixed effects, including both reduction and elevation in total protein levels, in Sitophilus spp. following treatment with anise oil, clove oil, and malathion. Additionally, Younes et al.^60^ observed increased protein levels in fourth-instar larvae of Trogoderma granarium treated with extracts of onion, garlic, rosemary, olive, peppermint, eucalyptus, and sunflower. Similarly, Khalaf et al.^61^ reported elevated protein content in Ephestia cautella following exposure to cumin, black seed, clove, ginger, and garlic oils.

### Total lipid content

Lipids represent the primary energy reserve in insects and are predominantly stored in the fat body rather than as glycogen. This mode of energy storage confers adaptive advantages, particularly in small insects, as lipids occupy less storage volume and yield more metabolic water upon oxidation compared with carbohydrates^62,63^.

The results summarized in Table 5 and depicted in Fig. 11 show that treatment with LC_50_ concentrations of the tested fixed oils and ethanolic extracts caused a significant increase in total lipid content in adult *A. gossypii*. The most substantial increases were recorded following treatment with *C. tiglium* oil and *U. dioica* extract, which elevated lipid levels by 47.61% and 33.33%, respectively, relative to the control.

These findings are in agreement with earlier reports indicating that plant-derived compounds may enhance lipid accumulation in insects. Abo El-Maaty^64^ observed increased lipid content in Trogoderma granarium treated with plant extracts, while^65^ reported similar effects in Musca domestica following exposure to aqueous plant extracts.

### Total carbohydrate content

Carbohydrates play a crucial role in insect metabolism, serving as a primary source of energy and as precursors for lipid and protein synthesis. Along with proteins and lipids, carbohydrates constitute the major classes of organic compounds essential for the structure and function of insect tissues.

The results presented in Table 5 and Fig. 11 demonstrate that exposure of adult *A. gossypii* to LC_50_ concentrations of the tested fixed oils and ethanolic extracts resulted in a significant decrease in total carbohydrate content compared with the control. The greatest reductions were observed following treatment with *C. tiglium* oil and *U. dioica* extract, which caused decreases of 40.00% and 26.66%, respectively.

These results are consistent with previous investigations reporting carbohydrate depletion in insects exposed to botanical compounds. Khalaf^66^ reported a significant reduction in carbohydrate content in *Muscina stabulans* treated with Rosmarinus officinalis and *Cymbopogon citratus* oils. Similar observations were made by Medhini et al.^67^ in Spodoptera littoralis and by Huron et al.^68^ in *Aphis craccivora*. Barakat^69^ documented decreased carbohydrate levels in adult *Myzus persicae* treated with volatile oil of *Ocimum basilicum* and fixed oil of Ricinus communis.

As well as the depletion of carbohydrates content in the treated insect larvae with both volatile and fixed oil may be related to inhibitions of carbohydrate hydrolyzing enzymes by treatment with plant oils. Protein is the major cell components which play the major role in all biological processes including reproduction development, growth and performance of its vital activities. The reduction in protein level may be a result of inhibition of DNA and RNA synthesis, low uptake of food and low assimilation of amino acids during the protein synthesis after the treatment of insecticides^69–73^.

Increase of the lipid content in the present work may be related to the effect of these plant extracts on the endocrine organs. According to, lipid accumulation in the fat tissues of one-day-old female desert locusts may be caused by high lipid deposition in the tissue combined with low lipid utilization, but it is more likely that lipid accumulation is directly related to a lack of juvenile hormone. The extracts of the examined plants may result in generation of corpora alata, which may enable an increase in the mean lipid content^74,75^.

Carbohydrates are generally of utmost importance because they can be used by an insect's body to produce energy or converse lipids or proteins. That explains why the overall amount of carbohydrates in the larvae's artificial diet decreased. More sugars may be metabolised to meet energy needs during stressful situations. This might be the cause of the decreased glucose levels in treated flies^76^.

Finally, the difference which induced in the biochemical activities of treated insects and its predator may be related to the variation in the phytochemical structure of plant extract due to the different toxicological effects of the existed different chemical constituents or functional groups as carboxylic acids, steroids, carbonyls, aliphatic amines (secondary and tertiary), cyclic ethers, phenols, aromatic rings, naphthenics and aliphatic (saturated and unsaturated) hydrocarbons.

The clustered heatmap (Fig. 11) provides a comprehensive visualization of the biochemical effects of the tested plant extracts and fixed oils on the adult stage of *Aphis gossypii*. Treatments and biochemical parameters were hierarchically clustered to reveal patterns of similarity and divergence. Notably, *C. tiglium* and *U. dioica* exhibited the most pronounced impact, causing substantial reductions in total protein and carbohydrate content, accompanied by marked increases in lipid levels. Conversely, *H. muticus* and *S. chinensis* induced moderate alterations across the measured parameters. The color gradient, ranging from deep red to deep blue, effectively highlights the magnitude and direction of change relative to the control, with red shades representing significant increases and blue shades indicating pronounced decreases. This visualization clearly demonstrates that the biochemical response of *A. gossypii* is strongly dependent on both the type of plant treatment and the specific metabolic parameter, providing a rapid and intuitive means to compare efficacy and mode of action among the tested botanical compounds.

The obtained LC_50_ values demonstrated promising insecticidal activity of the tested botanical materials under laboratory conditions; however, further field investigations are required to evaluate their practical applicability under natural environmental conditions. No visible phytotoxic symptoms were observed on the treated citrus leaves during the experimental period, indicating the relative safety of the tested materials toward the host plant. Moreover, the increase in total lipid content observed in treated aphids may reflect a physiological stress response or a compensatory metabolic mechanism associated with detoxification and energy storage under toxic stress conditions.

Plants play an important role in nature and in human life by produce natural chemical compounds that help protect them from insects and pests. These compounds can be used as natural insecticides when extracted. Many plants are also rich in antioxidants, which protect the body’s cells from damage caused by free radicals, helping to prevent various diseases^77,78^.

## Conclusion

The present study demonstrated that the tested botanical extracts and seed oils exhibit significant toxic and sub-lethal biochemical effects against adult *Aphis gossypii* under laboratory conditions. Among the evaluated treatments, *Croton tiglium* seed oil showed the highest insecticidal activity, followed by *Urtica dioica* extract, while *Simmondsia chinensis* oil exhibited the lowest toxicity. All tested materials induced marked alterations in the biochemical composition of treated aphids, including significant reductions in total soluble proteins and carbohydrates, accompanied by an increase in total lipid content, indicating disruption of normal metabolic processes.

These findings suggest that the evaluated plant-derived products act not only as toxic agents but also as metabolic disruptors, which may contribute to their overall insecticidal efficacy. Therefore, these botanical extracts represent promising eco-friendly alternatives for the management of *A. gossypii* and could be integrated into sustainable pest management programs. Further field studies are recommended to confirm their effectiveness under natural environmental conditions and to evaluate their safety on non-target organisms.

## Supplementary Information

Supplementary Information.

## Data Availability

All data supporting the findings of this study are available within the paper and its Supplementary Information.
