# Supplementary material for: Unveiling the toxicological and biochemical evaluation of selected botanical extracts against adult Aphis gossypii (Aphididae: Hemiptera)
Source: Sci Rep. 2026 Aug 21;16:26321. doi: 10.1038/s41598-026-66584-0 (PMC13498605; doi:10.1038/s41598-026-66584-0)
Supplement: Supplementary file 1 [file 41598_2026_66584_MOESM1_ESM.docx]

|  |  |
| --- | --- |
| **Fig. (S1):** GLC analysis of saponifiable components (fatty acids) of *Croton tiglum* fixed oil | **Fig. (S2):** GLC analysis of saponifiable components (fatty acids) of *Simmondsia chinensis* fixed oil. |

|  |  |
| --- | --- |
| **Fig. (S3):** GLC analysis of unsaponifiable components of *Croton tiglum* fixed oil. | **Fig. (S4):** GLC analysis of unsaponifiable components of *Simmondsia chinensis* fixed oil. |
